# Information on actual medication use and drug-related problems in older patients: questionnaire or interview?

**DOI:** 10.1007/s11096-016-0258-9

**Published:** 2016-02-01

**Authors:** Floor Willeboordse, Lucienne H. Grundeken, Lisanne P. van den Eijkel, François G. Schellevis, Petra J. M. Elders, Jacqueline G. Hugtenburg

**Affiliations:** Department of General Practice and Elderly Care Medicine, Institute for Health and Care Research, EMGO+, VU University Medical Center, Van der Boechorststraat 7, 1081 BT Amsterdam, The Netherlands; Netherlands Institute for Health Services Research, NIVEL, Po. Box 1568, 3500 BN Utrecht, The Netherlands; Department of Clinical Pharmacology and Pharmacy, VU University Medical Center, De Boelelaan 1117, 1081 HV Amsterdam, The Netherlands

**Keywords:** Actual medication use, Clinical medication review, Drug-related problems, Patient questionnaire, Polypharmacy, Netherlands

## Abstract

*Background* Information on medication use and drug-related problems is important in the preparation of clinical medication reviews. Critical information can only be provided by patients themselves, but interviewing patients is time-consuming. Alternatively, patient information could be obtained with a questionnaire. *Objective* In this study the agreement between patient information on medication use and drug-related problems in older patients obtained with a questionnaire was compared with information obtained during an interview. *Setting* General practice in The Netherlands. *Method* A questionnaire was developed to obtain information on actual medication use and drug-related problems. Two patient groups ≥65 years were selected based on general practitioner electronic medical records in nine practices; I. polypharmacy and II. ≥1 predefined general geriatric problems. Eligible patients were asked to complete the questionnaire and were interviewed afterwards. *Main outcome measure* Agreement on information on medication use and drug-related problems collected with the questionnaire and interview was calculated. *Results* Ninety-seven patients participated. Of all medications used, 87.6 % (95 % CI 84.7–90.5) was reported identically in the questionnaire and interview. Agreement for the complete medication list was found for 45.4 % (95 % CI 35.8–55.3) of the patients. On drug-related problem level, agreement between questionnaire and interview was 75 %. Agreement tended to be lower in vulnerable patients characterized by ≥4 chronic diseases, ≥10 medications used and low health literacy. *Conclusion* Information from a questionnaire showed reasonable agreement compared with interviewing. The patients reported more medications and drug-related problems in the interview than the questionnaire. Taking the limitations into account, a questionnaire seems a suitable tool for medication reviews that may replace an interview for most patients.

**Electronic supplementary material:**

The online version of this article (doi:10.1007/s11096-016-0258-9) contains supplementary material, which is available to authorized users.

## Impacts on practice

- Older patients report more medication and drug-related problems in an interview than a questionnaire, however there is reasonable agreement between the interview and questionnaire.
- A questionnaire seems a suitable tool that may be a surrogate for an interview for most patients and may increase the feasibility of conducting clinical medication reviews in daily practice.
- A questionnaire is available intended for use in general practice or pharmacy practices as preparation for a clinical medication reviews.

## Introduction

Older age is frequently accompanied with an increased prevalence of multiple chronic diseases, often resulting in the use of multiple medications or polypharmacy. Polypharmacy, usually defined as the chronic use of ≥5 prescribed medications, is associated with the occurrence of drug-related problems (DRPs) such as drug–drug interactions, inefficacy of treatment, adverse drug reactions (ADR), prescription errors and non-adherence [1]. A clinical medication review (CMR) can be used to detect potential DRPs and improve the quality of pharmacotherapy and patient outcomes [2–6]. A CMR is defined as a ‘structured, critical examination of the patient’s medicines with the objective of reaching an agreement with the patient about treatment, optimizing the impact of medicines, and minimizing the number of DRPs [7].

Preparing a CMR requires insight in actual medication use and knowledge of potential DRPs. Active patient participation is a prerequisite to determine how and which medications are actually used and to identify DRPs for successful medication reviews [3, 8, 9–11]. A gold standard for collecting patient-specific information on medication use and DRPs is not available. However, interviewing patients including a medication inspection, preferably during a home visit or the ‘brown bag’ method [12], seems the best method [13]. The Dutch guideline for polypharmacy in older patients recommends face-to-face interviews, however this is very time-consuming. [14, 15], Patient involvement in medication reviews is desirable and may improve patient outcomes, but as yet is not evidence-based and might not be needed at all times and costs [8].

Alternatively, a self-administered questionnaire could be used to obtain information from patients to conduct a CMR. Self-administered questionnaires are less time-consuming, can reach more people, provide standardized information and may be preferable for capturing sensitive topics in comparison to face-to-face interviews.

However, existing self-reported questionnaires on DRPs were not developed with the aim to obtain patient information relevant for CMRs [16, 17]. Interview protocols have been developed to support a CMR [13, 18]. In the present study a self-administered questionnaire was developed using an existing interview protocol and DRP classification system, to report actual medication use and DRPs from the patients’ perspective. We were interested in the agreement between the information obtained via the questionnaire and an interview and which patient groups showed better or worse agreement.

### Aim of the study

The aims of this study were: (1) to compare information on actual medication use and DRPs obtained by means of a questionnaire with information from a face-to-face interview and (2) to assess whether the extent of agreement for a number of patient and health characteristics differs between subgroups of patients.

### Ethical approval

The study was assessed by the Medical Ethics Committee of VU University Medical Centre (2011/408). In accordance with local regulatory guidelines and standards for Dutch human subjects protection (Medical Research Involving Human Subjects Act [WMO], 2005), this study proved to be exempt from further medical ethical review.

## Method

Information obtained by means of a questionnaire was compared with a face-to-face interview in 97 older patients with either polypharmacy or geriatric problems.

### Participants

Patients were recruited February–June 2013 from nine GP (general practitioner) practices in Haarlem, the Netherlands. Two patient groups aged ≥65 years were included: polypharmacy patients and patients with geriatric problems. Both groups were selected because there is no consensus on the best target group for medication reviews. Patients were identified based on information in the GP’s Electronic Medical Records (EMR) and the following criteria:

1. Polypharmacy was defined as the use of ≥5 chronic prescribed medications;
2. Geriatric problems were immobility, falls, dizziness, urine incontinence and impaired cognition [19]. Geriatric problems were identified on the basis of a selection of International Classification of Primary Care (ICPC) coded diagnoses [20] recorded in 2012 in the patients’ EMRs. To ensure that patients had an actual geriatric problem questions about current complaints were included in the questionnaire to be scored on a 3-point Likert scale; none, some or a lot problems (respectively 1, 2 or 3). Patients were included if they scored at least ‘2’ for at least one geriatric problem or if they reported ≥1 falls in the previous 6 months. Patients were eligible if they used ≥1 prescribed medication chronically.

Chronic medication use was defined as ≥3 prescriptions in the last 12 months recorded in the EMR.

Patients with a dementia diagnosis were not eligible. The GPs reviewed the list of eligible patients for exclusion criteria: terminally ill patients, recent severe psychiatric problems, or other personal issues making it not desirable to invite patients. Participants were asked to sign informed consent form. Patients who did not want to participate could return a no-participation form.

### Development of the questionnaire

A questionnaire to obtain information on actual medication use and DRPs to support CMR was developed on the basis of a previously developed interview to identify DRPs [18] and the Pharmaceutical Care Network Europe Classification for DRPs [21]. The questionnaire consisted of two parts; Part A: actual medication use and medication knowledge and Part B: patient experiences of DRPs (Electronic supplementary Material I). Ten experts (GPs, pharmacists, elderly care specialist, researchers) reviewed the questionnaire using a systematic scoring system in two rounds to obtain face- and content validity. All experts had content, textual and/or lay-out suggestions for the questionnaire and manual. In part A, five questions were deleted and one changed, in part B, two questions were deleted, two added and three were changed. The questionnaire was pilot-tested in two phases by seven and four patients, all ≥75 years using ≥8 medications recruited from one pharmacy. Each patient was asked to fill out the questionnaire in the presence of a researcher, who asked the patients to verbalise their thoughts (‘think aloud’). The patient’s behaviour was observed, such as skipping questions or hesitation. After the first round, the lay-out of part A on actual medication use was changed thoroughly and the sequence of questions of part B was changed. Following these revisions, a second pilot test confirmed that the questionnaire was suitable for older patients.

### Interview

Interviews were performed using a structured interview protocol by trained researchers using the same questions as the questionnaire. The interviewers received half a day of interview training and the first three interviews were conducted in pairs. To obtain information about actual medication use, the medication name, dosage and frequency were noted from the boxes and bottles, including any over the counter (OTC) medications. For each medication the patient was asked for the indication. A distinction was made between oral and non-oral medications based on ATC codes. There were eleven questions on DRPs and four main groups could be distinguished: possible adverse events, effectiveness, non-adherence, and user problems.

### Measurements

First, patients were asked to complete the questionnaire, second, patients were visited at home for an interview by a researcher. Information on gender, age, socio-demographics, self-perceived health status and geriatric problems was obtained from the questionnaire. Health literacy was measured using the REALM-D test, a score of ≤59 indicates low health literacy [22]. The number of chronic diseases per patient was calculated using the ICPC coded diagnoses in the EMRs based on a list of the most common chronic diseases in general practice [23].

### Statistical analyses

Descriptive statistics were used to describe patient characteristics. The agreement of the medication’s name and potential DRPs (dichotomous answers) between the questionnaire and the interview were presented in percentages and 95 % confidence intervals. Percentages were calculated for the reporting of medication and DRPs either only in the questionnaire or only in the interview. Agreement was assessed both at individual drug and DRP level as well as at patient level.

Independent *T* tests and Chi-square tests were performed to analyze differences in agreement in actual medication and DRPs for gender, age, living situation, education level, self-perceived health, health literacy, number of medications and number of chronic diseases.

Non-responder analyses were performed to detect differences in patient characteristics between participants and non-participants; descriptive and Chi-square statistics were used. All data was analyzed anonymously and carried out using IBM SPSS statistics version 20 software).

## Results

### Patient characteristics

**Fig. 1:**
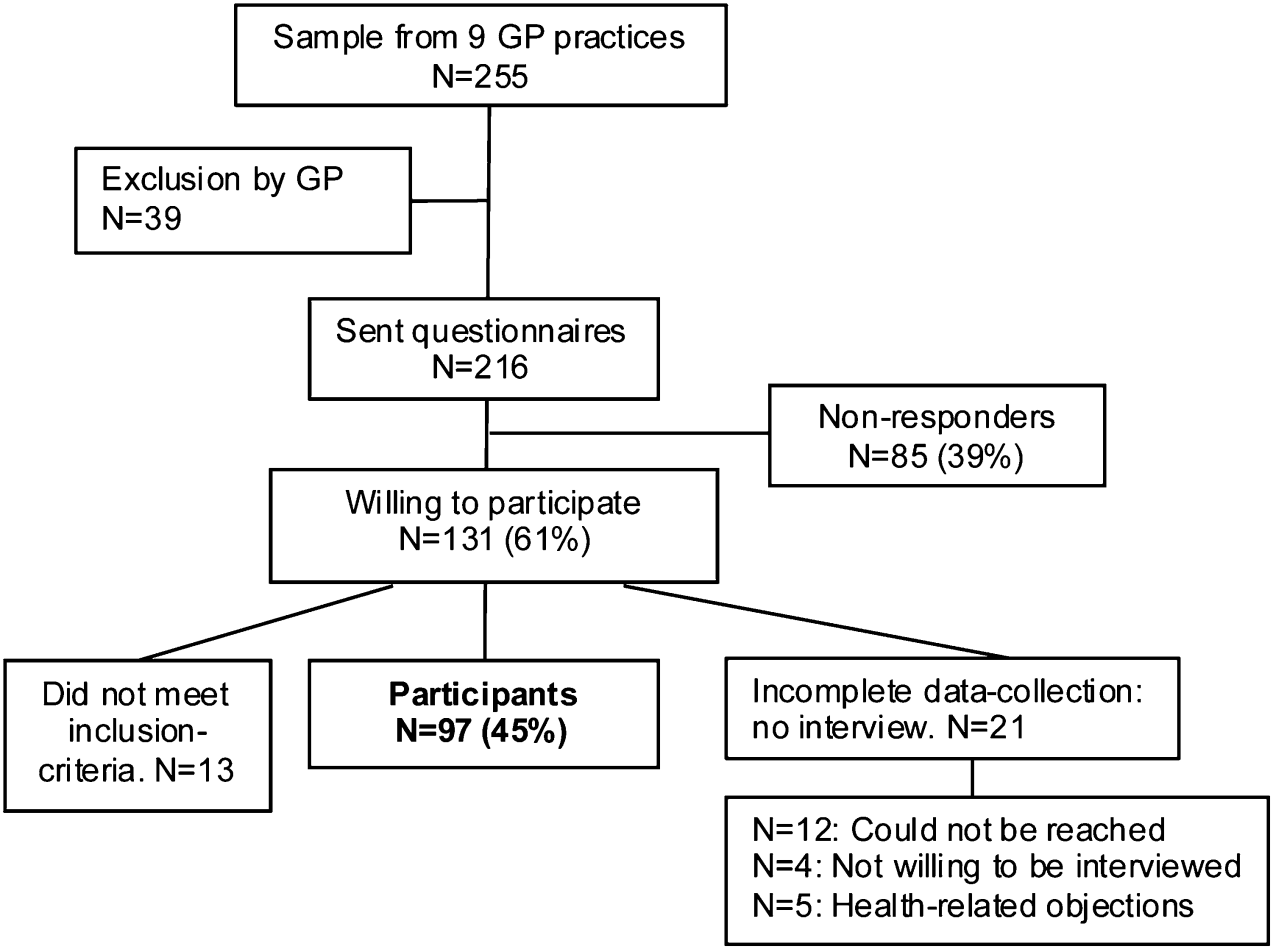
Flow diagram of participants

Of the 255 patients that were selected from the EMR records, 39 were excluded by the GP. Of the remaining 216 patients, 131 (61 %) were willing to participate in the study (Fig. 1). Complete data was obtained from 97 patients (44 polypharmacy patients and 53 geriatric problem patients). The mean duration of the interview was 16 [SD 7] min, excluding travelling and introduction time. The mean period between the receipt of the questionnaire and the interview was 9 [SD 5.2] days.

The mean age of the patients was 75.9 [SD 7.1] years and 72 % were women. The mean number of medications per patient according to the interview was 7.3 [SD 3.2] and 6.8 [SD 2.7] according to the questionnaire. The most common geriatric problems were mobility problems (73 %), followed by urine incontinence (50 %) and cognitive problems (40 %). Multimorbidity was common, the mean number of chronic diseases was 4.0 [SD 2.4]. 19 % of the patients had inadequate health literacy (Table 1).

**Table 1:** Patient characteristics

	Total
Patients, n	97
Women (%)	72 %
Mean age in years (SD) [range]	75.9 (7.1) [65–90]
Education level^a^	
Low %	53 %
Middle %	37 %
High %	10 %
Low health literacy^b^	18 (19 %)
Living alone	44 (45 %)
Mean no medications (SD) [range] in questionnaire	6.8 (2.7) [1–13]
Mean no medications (SD) [range] in interview	7.3 (3.2) [1–16]
% Reported ≥5 medications in either questionnaire or interview	80 (83 %)
Use of one or more OTC medications (%)	76 (88 %)
Self-perceived health	
Good to excellent	48 (50 %)
Fair to poor	49 (50 %)
Mean no chronic diseases (SD) [range]^c^	4.02 (2.4) [0–10]
Geriatric problems, n (%)	
Falling (≥1 last 6 months)	31 (32 %)
Mobility problems	71 (73 %)
Dizziness	33 (34 %)
Incontinence problems	48 (50 %)
Cognitive problems	39 (40 %)
Fear to fall	37 (39 %)

*SD* standard deviation, *OTC* over the counter

^a^Low education level: no education, primary education or first stage of basic education; middle education level: lower secondary education or second stage of basic education; high education level: upper secondary education or higher

^b^REALM score ≤59 [22]

^c^Chronic diseases according to set list [23]

### Comparison of participants and non-participants

Of the 85 patients not willing to participate, 27 indicated the reason for non-participation. Main reasons were no interest (N = 13), personal reasons/no reason (N = 11) or the use of few medications (N = 3). There were significantly more females among the participants (72 %), compared to non-participants (46 %). There were no significant differences between participants and non-participants in age and multimorbidity.

### Agreement on actual medication use

Table 2 shows the observed agreement on the level of medication and Table 3 on the patient level, which represents the agreement on the complete medication list.

**Table 2:** Agreement for medication use in questionnaire compared with interview at medication name level by patient characteristics

Patient characteristics	Agreement at medication name level (95 % CI)
All patients	87.6 % (84.7–90.5)
Gender	
Male	88.7 % (82.7–94.7)
Female	87.2 % (83.8–90.5)
Age	
<80 years	88.5 % (85.1–92.0)
>80 years	85.8 % (80.4–91.2)
Living situation	
Alone	87.7 % (83.7–91.6)
With partner	87.5 % (83.3–91.8)
Level of education	
Low	88.4 % (84.4–92.3)
Middle	87.1 % (82.6–91.6)
High	85.5 % (71.6–99.4)
Health literacy	
Low	83.5 % (76.6–90.4)
Adequate	88.6 % (85.3–91.9)
Self-perceived health	
Good to excellent	85.6 % (81.2–90.0)
Fair to poor	89.7 % (85.9–93.4)
Number of medications*	
<10	91.1 % (88.4–93.9)
≥10	78.4 % (71.9–84.9)
Chronic diseases	
0–3 chronic diseases	90.5 % (86.6–94.3)
≥4 chronic diseases	85.2 % (81.0–89.4)

*CI* confidence interval

* *p* value: <0.05

**Table 3:** Agreement for medication use in questionnaire compared with interview at patient level by patient characteristics (N = 97)

Patient characteristics	Complete medication list agreement at patient level (95 % CI)
All patients	45.4 % (35.8–55.3)
Gender	
Male	55.6 % (37.3–72.4)
Female	41.4 % (30.6–53.1)
Age	
<80 years	46.9 % (35.2–58.9)
>80 years	42.4 % (27.2–59.2)
Living situation	
Alone	43.2 % (29.7–57.8)
With partner	47.2 % (34.4–60.3)
Level of education	
Low	47.1 % (34.1–60.5)
Middle	41.7 % (27.1–57.8)
High	50.0 % (23.7–76.3)
Health literacy**	
Low	27.8 % (12.5–50.9)
Adequate	49.3 % (38.3–60.4)
Self-perceived health	
Good to excellent	40.8 % (28.2–54.8)
Fair to poor	50.0 % (36.4–63.6)
No of medications*	
<10	55.7 % (44.1–66.8)
≥10	18.5 % (8.2–36.7)
Chronic diseases**	
0–3 chronic diseases	54.5 % (40.1–68.3)
≥4 chronic diseases	37.7 % (25.9–51.2)

*CI* confidence interval

* *p* value: <0.05

** *p* value: <0.10

The total number of used medications according to the interview was 705, mean 7.3 [SD 3.2] per patient and according to the questionnaire 662, mean 6.8 [SD 2.7] per patient. The observed overall agreement was 87.6 % for all medications. Medications were more frequently mentioned only in the interview (8.8 %), than only in the questionnaire (3.3 %). The observed agreement for information on dosage and frequency was both 76 %. Of all medications reported, 12 % was non-oral. The agreement for non-oral medications was significantly lower than for oral medications (67.4 vs. 88.7 %).

Agreement of knowledge of medications indication was not assessed.

The agreement for patients using ≤10 medications was 91 % (95 % CI 88.4–93.9), significantly higher compared to 78 % (95 % CI 71.9–84.9) for patients using ≥10 medications (*p* < 0.001). There were no other significant differences in agreement on medication use between subgroups of patients (Table 2).

45.4 % of the patients had complete agreement for their total medication list (Table 3). There were no significant differences in agreement between subgroups based on gender, age, living situation, education level, or self-perceived health. The complete list agreement for patients using ≤10 medications was significantly higher (*p* = 0.01), 56 % compared to 18.5 % for patients using ≥10 medications. Participants with inadequate health literacy and ≥4 chronic diseases had a slightly lower complete list agreement (respectively 28 and 38 %) compared to participants with adequate health literacy and <4 chronic diseases (respectively 49 and 55 %), however no significant differences were found (both *p* = 0.099).

### Agreement on drug-related problems

The DRPs were categorized in adverse events, effectiveness problems, non-adherence, and user or practical problems (Table 4). There were more DRPs identified in the interview than with thequestionnaire, respectively 116 and 76 DRPs. The best overall agreement was found for adverse events and effectiveness problems, (78 and 79 %). For non-adherence and user problems the agreement was 71 and 68 %, respectively. For 31 % of all patients there was agreement for all DRPs.

**Table 4:** Observed agreement for drug-related problems

Drug-related problems	Prevalence questionnaire n (%)	Prevalence interview n (%)	Agreement % (95 % CI)	DRP only in questionnaire %	DRP only in interview %
Adverse events^a^	16 (17 %)	23 (24 %)	78.4 % (70.0–86.7)	7 %	14 %
Adherence^b^	25 (26 %)	41 (41 %)	71.1 % (60.8–79.4)	7 %	23 %
Effectiveness^c^	14 (14 %)	24 (25 %)	79.4 % (71.2–87.6)	5 %	16 %
User and practical problems^d^	21 (22 %)	28 (29 %)	68.1 % (58.6–77.4)	12 %	20 %

*DRP* drug-related problem, *CI* confidence interval

^a^Adverse events; self-reported suspected adverse drug events

^b^Effectiveness; doubts about the effect of the medications

^c^Adherence problems; either forgetting medications, under- and overuse or not taking medications

^d^User and practical problems; unable to use medications and practical problems

In total, 17 % of the patients reported to experience adverse events in the questionnaire and 24 % in the interview. Non-adherence problems were the most common DRP mentioned in the patient questionnaire and interview, respectively 26 and 41 %. Not all reported non-adherence problems may be serious, many patients reported to forget medicine(s) only once or twice per month. In total 23 % of the DRPs for non-adherence were only reported in the interview, compared to 7 % that was only mentioned in the questionnaire.

Effectiveness problems, defined as doubts about the effect of the medication by the patient, were also more frequently mentioned in the interview (25 %) than the questionnaire (14 %).

Finally, user and practical problems were also identified by both tools, 29 % in the interview and 22 % in the questionnaire. Patients indicated in the questionnaire that they had e.g. difficulties to using their medications due to fear of side effects (n = 3) or were experiencing practical problems such as the time of the day (n = 4) and difficulties with swallowing (n = 3). In the interview problems like opening a medication strip (n = 9) and difficulties with swallowing (n = 6) were the most frequently reported practical problems. The user and practical problems were in 20 % only reported in the interview, and 12 % was only reported in the questionnaire.

There were no significant differences in the agreement on DRPs between subgroups based on patient and health characteristics (results not shown). Most subgroups were too small for valid analyses. There were no significant differences for all covariates at patient level for total DRP agreement.

## Discussion

In this study we used a questionnaire on actual medication use and DRPs from the patient’s perspective as instrument for the use in daily practice of clinical medication reviews and compared this with face-to-face interviews. Information on actual medication use obtained by a patient interview had good agreement with information obtained by the questionnaire. There was complete agreement for the total medication list for almost half of the patients. For orally used medications there was better agreement between questionnaire and the interview as compared to non-oral medication. Agreement for DRPs was reasonable, the interview provided more information compared to the questionnaire.

In current guidelines face-to-face contact with the patient is recommended when preparing medication reviews. These activities are time-consuming and undermine the feasibility or implementation of medication review activities in daily GP and pharmacist practice. However, an overview of actual medication use is essential for medication reviews. It is known that GPs’ and pharmacists’ medication records and actual intake often mismatch [24, 25]. Results from an Australian study showed that medication use obtained by means of a telephone interview had good agreement with those obtained by means of an interview [26]. In this study agreement percentages were somewhat higher, up to 100 %, than ours. This suggests that more efficient tools, like a questionnaire or self-reports by phone, may be a surrogate for face-to-face interviews.

We were interested in differences in agreement between different subgroups according to patient and health characteristics. We found no differences in agreement on DRPs between subgroups. For actual medication use, there was a slightly better agreement for patients with fewer medications, fewer chronic diseases and adequate health literacy, suggesting that the use of a questionnaire is the best option for these patients. For a subgroup of vulnerable older patients and patients with limited (health) literacy a face-to-face interview is probably preferable. In addition, not all older patients will be able to fill out a questionnaire. However the good response rate indicates that many older patients are capable and willing to complete a questionnaire. Unfortunately, we could not trace whether the questionnaire was completed by the patients themselves or with support, however we know some patients were assisted by informal carers.

Dichotomous answers about the existence of DRPs were analysed. However, the preparation of a CMR requires additional qualitative information on DRPs for use in daily practice. This additional information may include signals for potential DRPs and their causes and can be addressed by the physician or pharmacist when discussing the results of a medication review.

Some limitations may have influenced the outcomes. First, the interviews were not performed by pharmacists or GPs, as the result, information might have been less complete. However, the interviews have been conducted by trained interviewers using a structured protocol. Second, there were relatively more women among the participants than among the non-participants, which might question their representativeness. However, a higher participation rate by women is common in healthcare and questionnaire research [27]. The similar age and number of chronic diseases between participants and non-participants and the good response rate (61 %) suggests good representativeness of the sample while female gender was not related to agreement levels. Third, the order of the questionnaire and the interview may have influenced the results. All patients started with the questionnaire which may partly explain that patients reported more medication and DRPs in the interview than the questionnaire. The effects of the different measuring methods cannot be distinguished from asking a second time similar questions by a different method. Finally, the GPs were asked to exclude patients for who an invitation for the study would not be desirable at this moment. In total 15 % of the sample was excluded. GPs may have excluded more vulnerable or complex patients, a target group for medication reviews, but may have more difficulties with written questionnaires. As stated above, this is a group of patients for which another approach appears more appropriate.

The questionnaire is intended for use in GP or pharmacy practices as preparation for a CMR, instead of more elaborate history taking. Information from the patient on actual medication use and potential DRPs clearly is the appropriate starting point for a CMR. Answers on the questionnaire can be particular signals to address in the CMR and requiring further exploration by questioning the patient. Since more information on its usefulness in practice is needed, the questionnaire is currently evaluated in an ongoing trial on CMR in elderly with geriatric problems [14].

## Conclusion

Overall, the information from the questionnaire showed reasonable agreement compared with the interview. Actual medication use as assessed from questionnaire data had good agreement with the interview-based assessment, especially for oral medication. Although more DRPs were identified by means of the patient interview than with the questionnaire, there was a reasonable agreement. Taking the limitations into account, a questionnaire seems a suitable tool to replace a face-to-face interview and may increase the feasibility and standardization of conducting CMRs in daily practice. More patients can be reached with a questionnaire and it is less time-consuming. However, for more vulnerable older patients an interview may be still needed.

## Electronic supplementary material

Below is the link to the electronic supplementary material.

Supplementary material 1 (DOCX 30 kb)
