# Supplementary material for: Information on actual medication use and drug-related problems in older patients: questionnaire or interview?
Source: Int J Clin Pharm. 2016 Feb 1;38:380–7. doi: 10.1007/s11096-016-0258-9 (PMC4828470; doi:10.1007/s11096-016-0258-9)
Supplement: Supplementary file 1 — Supplementary material 1 (DOCX 30 kb) [file 11096_2016_258_MOESM1_ESM.docx]

**Electronic Supplementary Material I. Translated questionnaire**

| **Part A**  Explanation:  We would like to know which medication you are using **the last month**, how often per day, how many and for what condition. Medications are tablets or capsules, but also (eye) drops, sprays, creams, drinks, inhaler puffs, suppositories etc.  You can use your medication overview from the pharmacy and the boxes or bottles from the pharmacy of the last month to fill in part A. Of course, you can ask somebody to help you with the questionnaire.  **The following questions you can fill in for each medication separately.**  **Medication 1.**  A. Name and dosage (in mg/g/ml): _______________________________________­­___  B. How often do you use this medication? ____________________ *per day / week / as needed*  C. How much do you use per time? *(e.g. 2 tablets)* _____________________________  D. What is this medicine for? ______________________________________________  **Medication 16.**  A. Name and dosage (in mg/g/ml): _______________________________________­­___  B. How often do you use this medication? ____________________ *per day / week / as needed*  C. How much do you use per time? *(e.g. 2 tablets)* _____________________________  D. What is this medicine for? ______________________________________________  Do you also use medications or supplements that you **purchase yourself** at e.g. the local drugstore? E.g. paracetamol, ibuprofen, vitamins, homeopathic or herbal medications?   - No - Yes            If yes, which ones and how often?  …………………………………………………………………………………………………………………………………….  …………………………………………………………………………………………………………………………………….  ……………………………………………………………………………………………………………………………………. |
| --- |

| **Part B**  In this part of the questionnaire we ask you about possible drug-related problems. If you want you can explain more about you answer.   1. **Do you experience in the last month any side effects due to you medications?**  - No 🡪 Continue with question 2 - Yes 🡪 Continue with the table below   If yes, which side effects did you experience?   \| **Side effect (type of complaint)** \| **By which medication(s)?**  If you do not know, fill in ‘?’ \| **Since how long do you have this complaint?** \| \| --- \| --- \| --- \| \| 1…………………………...... \| ……………………………… \| ……………………………… \| \| 2…………………………...... \| ……………………………… \| ……………………………… \| \| 3…………………………...... \| ……………………………… \| ……………………………… \|  1. **Are you worried about possible side effects of your medications??**  - No - Yes            If yes, which side effects from which medication(s) do you worry about?  …………………………………………………………………………………………………………………………………….   1. **Do you use medication(s) for which you have doubts that they really work for you?**  - No - Yes           If yes, for which medication(s)?  …………………………………………………………………………………………………………………………………….   1. **Did you forget to take one or more of your medications last month?**  - No - Yes   If yes, how often and for which medication(s)?  …………………………………………………………………………………………………………………………………….   1. **At what moments of the day you take your medication?**   (You can check multiple boxes)   - Before breakfast - During or after breakfast - During or after lunch - During or after diner - Before bedtime - As necessary - Otherwise, namely…………………………………………………………………………………………………………  1. **How do you take care not to forget your medication?**   (You can check multiple boxes)   - Use on regular times, such as before or after a meal - Pill box - Pre-packed bags per day/ medication-roll (Baxter), from the pharmacy - Alarm, phone reminder - Help of partner or family member - Help of home care or nurse - None of the above - Otherwise, namely,…………………………………………………………………………………………………….  1. **Did you, in the last month, intentionally skip or take less of a medication as prescribed?**  - No - Yes            If yes, which medication? And why did you skip or take less?  …………………………………………………………………………………………………………………………………….   1. **Did you, in the last month, intentionally take more of a medication as prescribed?**  - No - Yes   If yes, which medication? And why did you take more?  …………………………………………………………………………………………………………………………………….   1. **Did you, in the last month, stopped with a prescribed medication, without consulting the physician?**  - No - Yes   If yes, which medication? And why did you stop?  …………………………………………………………………………………………………………………………………….   1. **Do you know for all your medications how to use it?**  - No - Yes   If no, what would you like to know?  ………………………………………………………………………………………………………………………………   1. **Do you ever have difficulties to use your medications as you physician prescribed?**   (You can check multiple boxes)   - No - Yes, because of the multitude of medications……………………………….…….………………………………….. - Yes, because one or more of the medications are not effective for me…….……................................................ - Yes, because I do not know why I take the medications……………………………………………………………... - Yes, because I experience side effects……………………….………………………………………………………... - Yes, because I worry about possible side effects….…….…….…….…….…….…………………………………… - Yes, because I do not feel to take the medications.…….…….…….…….…….……………………………………. - Yes, because I forget to take the medications………..…….…….…….…….…….…………………………………. - Yes, because I cannot oversee and differentiate between all the different medications………………………….. - Yes, due to other reasons, namely……………….……………………………………………………………………. .……………………………………………………………………………………………………………………………………  1. **Do you ever have practical issues to use your medications?**   (You can check multiple boxes)   - No - Yes, because I have troubles with the times of the day………………………………………………….…………… - Yes, because I have difficulties with swallowing the tablet or capsule……..………………………….…………… - Yes, because I have difficulties with the medication strip or opening the package or bottle…………………….. - Yes, because I cannot read or understand the label on the medication package…………………….…………… - Yes, because the medication has a bad taste……………..………….………………………………….…………… - Yes, because I have difficulties to administer the medication(s) (e.g. inhaler, eye drops)……………………….. - Yes, due to other reasons, namely ……………………………………………………………………………………..   ……………………………………………………………………………………………………………………………………   1. **Do you have any additional comments, problems. questions, or preferences about your medications or health?**   …………………………………………………………………………………………………………………………………..  …………………………………………………………………………………………………………………………………..  …………………………………………………………………………………………………………………………………..  …………………………………………………………………………………………………………………………………..  …………………………………………………………………………………………………………………………………..  …………………………………………………………………………………………………………………………………..  …………………………………………………………………………………………………………………………………..  …………………………………………………………………………………………………………………………………..  …………………………………………………………………………………………………………………………………..  ………………………………………………………………………………………………………………………………….. |
| --- | --- | --- | --- | --- | --- | --- | --- | --- | --- | --- | --- | --- |
